# Fecal microbiota in children with juvenile idiopathic arthritis treated with methotrexate or etanercept

**DOI:** 10.1186/s12969-021-00542-0

**Published:** 2021-04-26

**Authors:** Anders Öman, Johan Dicksved, Lars Engstrand, Lillemor Berntson

**Affiliations:** 1 Department of Women’s and Children’s Health, Uppsala University, Uppsala, Sweden; 2 Department of Animal Nutrition and Management, Swedish University of Agricultural Sciences, Uppsala, Sweden; 3 Center for Translational Microbiome Research, CTMR, Department of Microbiology, Tumor and Cell Biology (MTC), Karolinska Institutet, Science for Life Laboratory, Solna, Sweden

**Keywords:** Arthritis, juvenile, Gastrointestinal microbiome, Short-chain fatty acids, Methotrexate, Etanercept

## Abstract

**Background:**

Alterations in the composition of the fecal microbiota in children with juvenile idiopathic arthritis (JIA) have been observed in several studies, but it has not been determined whether the standard treatment for JIA changes the composition or function of the microbiota.

The first-line disease-modifying anti-rheumatic drug for treatment of JIA is usually methotrexate, followed or supplemented by anti-tumor necrosis factor alpha drugs, such as etanercept. The aim of this study was to investigate the effects of methotrexate and etanercept treatments on the fecal microbiota and the fecal short-chain fatty acids (SCFAs) in children with JIA.

**Methods:**

In this multicenter study, the composition of fecal microbiota from 45 treatment-naïve children with JIA was compared with that from 29 children treated with methotrexate and 12 children treated with etanercept. We also made pairwise comparisons of 15 children sampled before and during methotrexate treatment and 7 children sampled before and during etanercept treatment.

The microbiota was determined using sequencing amplicons from the V3 and V4 regions of the 16S rRNA gene. Alpha-diversity, community composition, and relative abundances of bacterial taxa were analyzed in all comparisons. Analyses of fecal SCFAs, using a high-performance liquid chromatograph, were performed for the pairwise comparisons.

**Results:**

We did not find any significant differences in α-diversity or community composition of microbiota. However, principal coordinate analysis indicated a change in community composition in 7 of the 15 paired samples before and during methotrexate and 2 of the 7 paired samples before and during etanercept.

Comparisons of the relative abundance of taxa revealed minor differences before and during treatment with methotrexate or etanercept, but they were not significant after correction for multiple analyses, and the unpaired and paired analyses did not show similar changes.

There were no significant differences in levels of fecal SCFAs before and during treatment with methotrexate or etanercept.

**Conclusions:**

Treatment with methotrexate or etanercept had minor, but no significant or consistent changes either on composition of microbiota or on levels of SCFAs, suggesting that these changes are not related to the therapeutic effects of methotrexate or etanercept.

## Background

Disturbances of the intestinal microbiota have been connected to several autoimmune diseases [1] and alterations in the composition of the fecal microbiota in children with juvenile idiopathic arthritis (JIA) have been observed in several studies [2–7]. Factors that can affect the intestinal microbiota, such as exposure to antibiotics [8], shorter breastfeeding [9] and delivery by cesarean section [10] have also been linked to an increased risk for JIA.

It has been speculated that the disease course of JIA could be affected through modification of the intestinal microbiota, but it has not been determined whether the standard treatment for JIA leads to any changes in the composition or function of the microbiota.

The first-line disease-modifying anti-rheumatic drug (DMARD) for treatment of JIA is usually methotrexate (MTX), followed or supplemented by anti-tumor necrosis factor alpha (anti-TNF-α) drugs, a group of the so-called biological DMARDs (bDMARDs). Methotrexate is a folate pathway antagonist, inhibiting dihydrofolate reductase when given at high doses for treatment of leukemia, but when given at lower doses, for treatment of rheumatic diseases, other mechanisms are probably involved [11]. The folate pathway also exists in microbes and treatment with MTX might thereby affect the intestinal microbiota. Gastrointestinal side effects are very common during MTX treatment [12] and some studies have shown a lower α-diversity and changes in the relative abundance of intestinal microbiota in mice treated with MTX [13, 14].

TNF-α is a pro-inflammatory cytokine involved in the pathogenesis of several inflammatory diseases, including JIA, and anti-TNF-α treatment has been very successful in children with JIA. Etanercept (ETN) has been the most common anti-TNF-α drug used in children with JIA thus far. In a study on mice with proteoglycan-induced arthritis, treatment with ETN restored an increased gut permeability and changed the microbiota composition to one more similar to that in control mice [15]. On the other hand, a large German register study has shown an increased risk for inflammatory bowel disease (IBD) in children with JIA treated with ETN as monotherapy [16]. This increases the indication for studying the impact of ETN treatment on the gut microbiota in children with JIA.

The effects of MTX and ETN on gut microbiota in humans have been studied very scarcely. In patients with rheumatoid arthritis (RA), one study showed that treatment with DMARDs, including MTX, partly modified the gut microbiome [17], but in another study the treatment was not associated with any obvious changes in the gut microbiota when compared with that in treatment-naïve patients [18]. However, in the same study, treatment with ETN affected the gut microbiota in a way that was described as beneficial, with an increase in the abundance of the phylum *Cyanobacteria*, and also in *Nostocophycideae* and *Nostocales*, while *Deltaproteobacteria* and *Clostridiaceae* decreased.

Many studies have focused mainly on the composition of intestinal microbiota, but most likely it is just as important to study the function of the microbiota and its association to the immune system, for example by exploring the levels of short-chain fatty acids (SCFAs). SCFAs, such as acetate, butyrate, propionate and valerate, are produced by the colonic microbiota during active fermentation of soluble dietary fibers and starches in the large intestine [19]. The SCFAs produced by the microbiota have been linked to several effects on the mucosal immune system in humans and in mice [20–22]. In particular, the production of butyrate has been shown to have a positive effect on joint inflammation in mouse models [23–25].

The aim of this study was to investigate the possible effects of MTX and ETN treatments on fecal microbiota composition and production of SCFAs in children with JIA. As far as we know, this has not been studied earlier.

## Methods

### Study participants

In this multicenter study, 86 children with JIA were included from four Swedish counties (Uppsala, Dalarna, Gävleborg and Västmanland), and a total of 108 fecal samples were collected from these children. The inclusion of study participants was carried out between 2012 and 2017 and the children with JIA were categorized based on the criteria established by the International League of Associations for Rheumatology [26]. Exclusion criteria for the children with JIA were other autoimmune diseases, including IBD, the use of antibiotics within the last 6 months before sampling, and special diet because of intolerance. Of the 86 children with JIA who were included in the study, 45 children were treatment-naïve at the time of sampling, 14 children were on single treatment with MTX, 5 children were on treatment with ETN, 15 children were sampled both as treatment-naïve and during single treatment with MTX and 7 children were sampled both as treatment-naïve and during treatment with ETN.

The use of non-steroidal anti-inflammatory drugs (NSAIDs) was investigated retrospectively for the participants, by reviewing their medical records. In the group of 45 children with JIA not treated with MTX or ETN at the time of sampling, nine were on continuous treatment with NSAIDs (of whom two were also treated with proton-pump inhibitors (PPI)), nine had been treated with NSAIDs occasionally before sampling, and 17 had not been treated with NSAIDs for at least 1 month before sampling. For 10 children, information about NSAID use was not available.

Only three children were treated with NSAIDs at the same time as they were treated with MTX (one of whom was also treated with PPI), and none of the children were treated with NSAIDs at the same time as they were treated with ETN. For five children on MTX and one child on ETN, information about NSAID use was not available.

### Collection of fecal samples

The participants collected fecal samples at home and stored them at + 4 °C until they were delivered to the hospital, which had to be done within a maximum of 60 h after sampling. When the samples arrived at the hospital, they were initially placed in − 25 °C freezers before being moved to − 70 °C freezers, where they were stored until analyses.

### DNA extraction, amplification and sequencing

For each analysis, 30–100 mg of fresh frozen fecal sample was used, and purified DNA was extracted as previously described [27]. For the 1-step polymerase chain reaction amplification, 50 ng of template DNA was used, with primers targeting the 341f-805r region of the 16S rRNA gene [27]. Finished libraries were quantified using Invitrogen’s QuantIt fluorometric assay (Thermo Fischer Scientific, Waltham, Massachusetts, USA) before being pooled to equimolar amounts and sequenced in parallel to whole bacterial genomes in a MiSeq instrument (Illumina Inc., San Diego, CA, USA) using 2 × 300 base pair reads and V3 chemistry.

### Sequencing quality control and amplicon sequence variant picking

Raw sequencing reads were pre-processed with Cutadapt to remove primer sequences, low-quality (< 15) 3′-bases and sequences containing any N. The resulting reads were submitted to DADA2 [28] for merging, amplicon sequence variant picking and taxonomy assignment based on the SILVA 128 database, as described earlier [29].

### Analyses of short-chain fatty acids

The quantifications of SCFAs were performed using a high-performance liquid chromatography machine from Agilent technology 1100 series (Agilent Technologies, Inc., Santa Clara, USA).

### Data comparisons

We compared the fecal microbiota from 29 children sampled during single treatment with MTX and 12 children sampled during treatment with ETN (eight on a combination with MTX) with the fecal microbiota from the 45 treatment-naïve children with JIA.

Twenty-two of the children sampled during treatment had also been sampled before the start of treatment, when they were treatment-naïve, making pairwise comparisons possible in 15 children treated with MTX and 7 treated with ETN. Three of the children treated with ETN were on combined treatment with MTX.

To examine if anti-inflammatory monotherapy with NSAIDs had an impact on the results, the fecal microbiotas from nine children with JIA treated continuously with NSAIDs were compared with the fecal microbiotas from 17 untreated children with JIA.

Alpha-diversity, community composition of microbiota and relative abundances of bacterial taxa were analyzed in comparisons between all groups. Analyses of relative abundances were performed at the taxonomic levels of phyla, families and genera.

Analyses of SCFAs in the fecal samples were performed for the pairwise comparisons of children sampled both before and during treatment.

### Statistics

Patient characteristics and time courses were described using median and interquartile range or total number and percent of study cohort.

Nonparametric tests were used for calculations of microbial data, since the data did not follow a normal distribution.

Comparisons of α-diversity and community composition were made using all amplicon sequence variants. For the calculations of relative abundance of taxa, only the taxa present in > 25% of the samples and with a mean abundance of > 0.1% were analyzed.

Measures of α-diversity in the fecal samples were calculated using the Chao-1 index and the Shannon diversity index. These indices were compared between treated and untreated children, using a logistic regression model with age at sampling as a covariate. For pairwise analyses of children sampled before and during treatment, the Wilcoxon signed-rank test was used.

The community composition of the microbiota was investigated by creating principal coordinate analysis (PCoA) plots for visual comparisons, and the analysis of similarity (ANOSIM) test was applied for calculations. The PCoA plots were based on Bray Curtis distances.

Analyses for relative abundances of taxa were performed at three taxonomic levels (phyla, families and genera). Logistic regression with age as covariate was used for calculations of differences between treated and untreated children, while the Wilcoxon signed-rank test was used for pairwise comparisons of fecal samples from children sampled both before and during treatment.

The Wilcoxon signed-rank test was used for the pairwise comparisons of SCFAs.

The Statistical Package for Social Sciences, version 26 (SPSS Inc., Chicago, IL, USA) was used for calculations of the logistic regression models and for the Wilcoxon signed-rank tests. Calculations of the Chao-1 index, the Shannon diversity index, ANOSIM and PCoA plots were carried out using the Paleontological Statistics software, version 3.22 [30].

*P* values < 0.05 were considered statistically significant. The Benjamini-Hochberg procedure was used to correct for multiple comparisons and the false discovery rate was set to 0.05 [31].

## Results

Clinical features for the treatment-naïve children with JIA and the children with JIA sampled during treatment with MTX or ETN are presented in Table 1. Clinical features for the children with JIA, with paired samples before and during treatment with MTX or ETN are presented in Table 2.

**Table 1:** Characteristics of 86 children with JIA, either treatment-naïve, or treated with MTX or ETN

	Treatment-naïve (***n*** = 45)	MTX (***n*** = 29)	ETN (***n*** = 12)
Female sex (%)	25 (56)	19 (66)	6 (50)
Age at sampling, years	10.9 (5.1–13.6)	9.3 (5.7–11.8)	8.22 (5.7–13.8)
Time from JIA onset to sampling, years	0.4 (0.2–3.1)	1.7 (1.0–3.8)	1.6 (1.0–2.6)
Time from start of medication to sampling, years		0.5 (0.3–0.8)	0.3 (0.2–0.7)
JIA category at onset, no (% girls)			
Oligoarticular	29 (62)	14 (71)	3 (67)
Polyarticular RF neg	5 (60)	11 (73)	5 (60)
Polyarticular RF pos	0	0	1 (100)
Enthesitis-related	7 (29)	1 (0)	2 (0)
Psoriatic	1 (0)	0	0
Undifferentiated	3 (67)	3 (33)	1 (0)

Data are median (1st–3rd quartile), unless otherwise indicated

*JIA* Juvenile idiopathic arthritis, *MTX* Methotrexate, *ETN* Etanercept, *RF* Rheumatoid factor

**Table 2:** Characteristics of 22 children with JIA, sampled both when treatment-naïve and during treatment

	Treatment-naïve (***n*** = 15)	MTX (***n*** = 15)	Treatment-naïve (***n*** = 7)	ETN (***n*** = 7)
Female sex (%)	11 (73)		2 (29)	
Age at sampling, years	9.1 (4.1–11.0)	9.2 (4.6–11.1)	13.0 (8.0–14.9)	13.4 (8.5–15.8)
JIA onset to sampling, years	0.4 (0.1–1.7)	1.2 (0.8–2.2)	0.34 (0.3–1.3)	2.1 (1.0–2.7)
Start of medication to sampling, years		0.5 (0.3–0.7)		0.3 (0.2–0.3)
JIA category at onset, no (% girls)				
Oligoarticular		7 (86)		1 (100)
Polyarticular RF neg		6 (67)		2 (0)
Polyarticular RF pos		0		1 (100)
Enthesitis-related		1 (0)		2 (0)
Undifferentiated		1 (100)		1 (0)

Data are median (1st–3rd quartile), unless otherwise indicated. Fifteen children were on single treatment with MTX, and 7 children were on ETN, as single treatment or in combination with MTX, at second sampling

*JIA* Juvenile idiopathic arthritis, *MTX* Methotrexate, *ETN* Etanercept, *RF* Rheumatoid factor

The mean number of sequences obtained from the fecal samples was 23,058, with a range from 5024 to 60,580. There were no large differences in numbers of sequences between the groups. The mean number of sequences obtained from the 45 treatment-naïve children, used for comparisons to children on MTX or ETN, was 22,985, while the mean number of sequences for children treated with MTX or ETN were 22,943 and 26,165, respectively.

Relative abundances of taxa were used for all analyses, and differences in sequence counts between samples were thereby equalized. The most abundant phyla, calculated as means from all samples, were *Firmicutes* (56%), *Bacteroidetes* (35%), *Proteobacteria* (6%) and *Actinobacteria* (2%).

### Comparisons between children treated with MTX or ETN and treatment-naïve children

Analyses with Chao-1 index and Shannon diversity index did not show any significant differences in α-diversity between children treated with MTX or ETN and treatment-naïve children (data not shown).

**Fig. 1:**
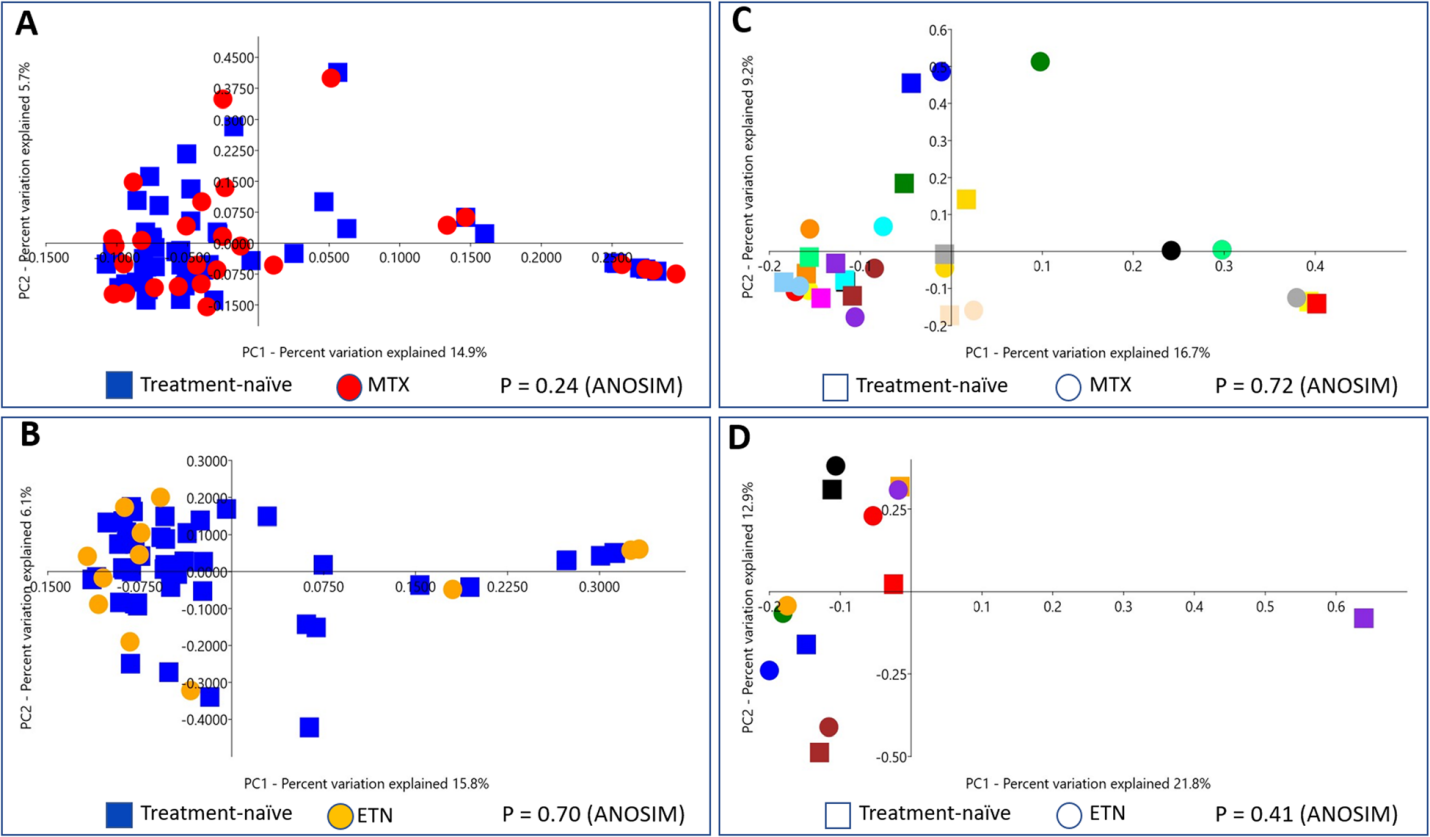
PCoA analyses of microbial community composition in fecal samples from children with JIA. The samples are clustered in the PCoA plots based on similarities in bacterial composition. Squares represent samples from treatment-naïve children and circles represent samples from children during treatment with MTX or ETN. **a**. PCoA plot for 45 treatment-naïve children and 29 children treated with MTX. **b**. PCoA plot for 45 treatment-naïve children and 12 children treated with ETN. **c**. PCoA plot for paired analyses of 15 children sampled both when treatment-naïve and later during treatment with MTX. The figures for each individual child have the same color for both the sample collected when treatment-naïve and the sample collected during treatment with MTX. **d**. PCoA plot for paired analyses of 7 children sampled both when treatment-naïve and later during treatment with ETN. The figures for each individual child have the same color for both the sample collected when treatment-naïve and the sample collected during treatment with ETN. JIA = juvenile idiopathic arthritis, PCoA = principal coordinates analysis, MTX = methotrexate, ETN = etanercept, ANOSIM = analysis of similarity

Principal coordinate analysis was used to investigate if the microbial community composition differed between the treated and the treatment-naïve children. The analysis did not reveal any separate clustering, either for children treated with MTX (Fig. 1a) or children treated with ETN (Fig. 1b), indicating that these therapies did not alter the microbiota composition. The visual analysis with PCoA was also confirmed with ANOSIM, which did not show any significant differences between treated and treatment-naïve children (*p* = 0.28 for MTX, *p* = 0.70 for ETN). Comparisons of the relative abundance of taxa revealed a few results which differed between children on treatment and treatment-naïve children, before adjustment for multiple analyses. In children treated with MTX, there was a higher relative abundance of *Subdoligranulum*, and in children treated with ETN, there was a higher relative abundance of *Lachnospiraceae NK4A136 group,* compared with in treatment-naïve children. However, none of the results were significant after correction for multiple analyses, and the standard errors for the results were large compared with their means (Table 3).

**Table 3:** Differences in relative abundances of taxa between treatment-naïve children and children treated with MTX or ETN

Taxa influenced by medical treatment^**a**^	Treatment-naïve (***n*** = 45)	MTX (***n*** = 29)	ETN (***n*** = 15)	Crude ***p*** value*	Adjusted ***p*** value**
**MTX:**					
*Subdoligranulum* (G)	0.028 (0.023)	0.065 (0.067)		0.005	0.27
**ETN:**					
*Lachnospiraceae_NK4A136_group* (G)	0.016 (0.015)		0.031 (0.033)	0.04	1

^a^Selected list of taxa with crude *p* value < 0.05. Data are mean (SE), unless otherwise indicated

*P values calculated using logistic regression with age at sampling as a covariate

**False discovery rate control using the Benjamini-Hochberg procedure

*JIA* Juvenile idiopathic arthritis, *MTX* Methotrexate, *ETN* Etanercept, *RF* Rheumatoid factor, *F* Family, *G* Genus, *SE* Standard error of the mean

### Pairwise comparisons of samples before and during treatment with MTX or ETN

Pairwise comparisons of samples collected from the same children both before and during treatment with MTX or ETN showed no significant changes in α-diversity (data not shown).

In the PCoA plot for children with paired samples before and during treatment with MTX (Fig. 1c), eight of the children had paired samples that clustered close together, indicating a similar composition of microbiota before and after treatment. For seven of the children, the paired samples were clearly separated from each other, indicating a change in microbiota composition between the two sampling sessions. Five of the seven children had a similar shift, where the first samples, collected when untreated, clustered with the other eight pairs in the plot, but the other two children had an opposite shift. ANOSIM showed no significant differences between samples before and during MTX treatment (*p* = 0.72), but that analysis did not take into account that the samples were paired.

In the PCoA plot for children with paired samples before and during treatment with ETN (4 out of 7 in combination with MTX) (Fig. 1d), there was a large cluster including 12 of the 14 samples. The last two samples clustered together, but separately from the other 12. The paired samples for five of the seven children clustered close together, indicating a similar community composition both before and during treatment. For two children, the paired samples were situated far away from each other in the plot, indicating a change in community composition. Both of these children had a similar shift in community composition, where their first samples, as untreated, were clustered together far away from all other samples in the plot, and their second samples, during treatment, were located in the large cluster together with the samples from the other five pairs. Both of these children were boys diagnosed with the JIA category enthesitis-related arthritis, one of them treated with ETN as single treatment and one of them treated with ETN and MTX in combination. These two children did not differ from the other children regarding age at sampling, time between the two samples or time from disease onset until first sampling.

ANOSIM showed no significant difference (*p* = 0.41) between samples before treatment and samples during treatment.

Comparisons of the relative abundance of taxa in samples before and during treatment with MTX or ETN showed lower relative abundances of *Rikenellaceae*, *Veillonellaceae*, *Bacteroidales_S24-7_group*, *Alistipes* and *Prevotellaceae_NK3B31_group* during treatment with MTX, before adjustment for multiple analyses (Table 4), but no differences in the relative abundances of taxa before and during treatment with ETN. None of the mentioned differences before and during treatment with MTX were significant after correction for multiple analyses.

**Table 4:** Differences in relative abundances of taxa in children sampled when treatment-naïve and during treatment with MTX or ETN

Taxa influenced by medical treatment†	Treatment-naïve	On medical treatment	Crude ***p*** value*	Adjusted p value**
**MTX in 15 children:**				
*Rikenellaceae* (F)	0.043 (0.023)	0.029 (0.024)	0.02	0.53
*Veillonellaceae* (F)	0.017 (0.019)	0.011 (0.014)	0.03	0.36
*Bacteroidales_S24-7_group* (F)	0.005 (0.009)	0.002 (0.006)	0.04	0.33
*Alistipes* (G)	0.043 (0.023)	0.028 (0.024)	0.02	1
*Prevotellaceae_NK3B31_group* (G)	0.031 (0.068)	0.009 (0.026)	0.04	1
**ETN in 7 children:**				
No significant differences	–	–	–	–

^**†**^Selected list of taxa with crude p value < 0.05. Data are mean (SE), unless otherwise indicated

*P values calculated using Wilcoxon signed-rank test

**False discovery rate control using the Benjamini-Hochberg procedure

*JIA* Juvenile idiopathic arthritis, *MTX* Methotrexate, *ETN* Etanercept, *RF* Rheumatoid factor, *F* Family, *G* Genus, *SE* Standard error of the mean

For the pairwise comparisons, we also analyzed the amounts of SCFAs in the fecal samples. There were no significant differences in the amounts of acetate, butyrate, propionate or valerate in any of the pairwise analyses of children with samples collected both before any treatment and during treatment with MTX or ETN, but analyses did show a higher level of iso-butyrate during treatment with MTX compared with before any treatment (*p* = 0.02; Table 5). However, iso-butyrate occurred at very low levels in all samples.

**Table 5:** Fecal SCFAs in children sampled when treatment-naïve and during treatment with MTX or ETN

SCFAs (mmol/L)	Treatment-naïve (***n*** = 15)	MTX (***n*** = 15)	p value*	Treatment-naïve (***n*** = 7)	ETN (***n*** = 7)	***P*** value*
Acetate	58.6 (36.9)	49.1 (26.0)	0.57	47.5 (25.1)	43.2 (18.4)	0.61
Propionate	14.4 (7.0)	12.4 (7.0)	0.50	13.9 (8.0)	15.3 (9.1)	0.74
Isobutyrate	1.6 (1.0)	2.1 (1.4)	0.02	3.0 (4.1)	1.1 (0.6)	0.75
Butyrate	17.7 (13.9)	15.0 (10.9)	0.53	14.5 (11.8)	17.1 (10.8)	0.61
Valerate	4.3 (3.0)	4.6 (2.7)	0.44	4.8 (2.2)	5.7 (2.7)	0.50

Data are mean (SE), unless otherwise indicated

*Wilcoxon signed-rank test

*SCFAs* Short-chain fatty acids, *MTX* Methotrexate, *ETN* Etanercept, *SE* Standard error of the mean

### Comparisons between children treated with NSAIDs and children without NSAIDs

Analyses with the Chao-1 index and the Shannon diversity index did not show any significant differences between children during treatment with NSAIDs and untreated children. A PCoA plot did not show any clustering and calculations with ANOSIM showed no differences in community composition (*p* = 0.31).

Univariate comparisons showed a lower relative abundance of *Parabacteroides* in fecal samples from children during treatment with NSAIDs (*p* = 0.04), but the result was not significant after correction for multiple analyses.

## Discussion

In the present study, treatment with MTX or ETN did not show any significant or consistent impact on the composition of microbiota or the levels of SCFAs in fecal samples from children with JIA.

This is the first study on a possible influence of MTX and ETN treatment on the composition of fecal microbiota in children with JIA, but two earlier studies, by Zhang et al. and Picchianti-Diamanti et al., have examined the effect of MTX on fecal microbiota in adults with RA [17, 18]. In the study by Picchianti-Diamanti et al., the effect of ETN on fecal microbiota was also studied [18]. The study by Zhang et al. showed that treatment with MTX partially modified the gut microbiome in adults with RA, to a composition more similar to that in healthy adults. They did not examine microbial diversity or relative abundances of taxa during treatment, and their results are thus not comparable to the results of the present study. In the study by Picchianti-Diamanti et al., eleven patients treated with MTX were compared with eleven treatment-naïve patients, and the only significant difference was a lower abundance of *Enterobacteriales* (*p* = 0.05) in treated patients. In the same study, ten patients treated with ETN were compared with eleven treatment-naïve patients, showing an increase in the *Nostocales* order (*p* = 0.03) and decreases of *Deltaproteobacteria* and *Clostridiaceae* (*p* = 0.05 for both) in patients treated with ETN. However, *Nostocales* was rare, detected in only four of the ten patients with ETN (relative abundance 0.35%) and in none of the treatment-naïve patients in the study by Picchianti-Diamanti et al. Also, none of the results were corrected for multiple analyses. Analyses of fecal microbiota, using 16S-rRNA gene sequencing, generate sequence reads for several hundreds of bacterial taxa at different taxonomic levels, and since the knowledge about which taxa that might be linked to different diseases is limited, researchers have to make comparisons involving large numbers of different taxa. The risk of type I errors is high in such repeated comparisons and correction for multiple testing should be used in most cases [32].

In the present study, comparisons between children with JIA treated with MTX as single treatment and treatment-naïve children showed a higher abundance of *Subdoligranulum* during treatment. In the paired analyses where children with JIA were sampled before any treatment and later sampled again during treatment with MTX, the five taxa *Rikenellaceae*, *Veillonellaceae*, *Bacteroidales_S24-7_group*, *Alistipes* and *Prevotellaceae_NK3B31_group* showed lower abundances in samples during treatment with MTX. However, none of the mentioned findings were significant after correction for multiple analyses, nor were these differences consistent in the unpaired and paired analyses. Thus, no obvious impact of MTX on the relative abundance of taxa in the gut microbiota could be seen.

The PCoA analysis of the paired samples, collected before and during treatment with MTX, showed that 7 out of 15 children had a shift in the community composition of fecal microbiota during treatment. The other eight children showed a very similar community composition in samples collected before and during MTX. The gut microbiota is a complex system with many different bacteria and it may be that significant changes in a few specific bacteria are less important than small, insignificant, changes in many bacteria with similar properties, which can be detected in analyses of community composition. The shift in community composition for seven children could possibly indicate that MTX has an effect on the composition of gut microbiota in some individuals. Other factors may affect which children get a changed bacterial composition during treatment, but we did not find any characteristics that differed between the two groups, in terms of JIA category, age at sampling, time from JIA onset to the first sampling or time on treatment. Also, the shift in community composition during treatment did not exhibit a similar pattern for all the seven children. In addition, neither the PCoA plot nor ANOSIM showed any differences in community composition in the unpaired comparisons between children treated with MTX and treatment-naïve children.

Comparisons of children with JIA treated with ETN and treatment-naïve children showed no significant differences in α-diversity, community composition or relative abundance of taxa. Neither did the analyses of α-diversity or relative abundance of taxa in the paired analyses of children sampled before and during treatment with ETN. However, in the PCoA plot for paired samples, collected before and during treatment with ETN, two of the seven pairs showed a clear shift in community composition between samples collected before treatment and samples collected during treatment. The two samples collected before any treatment were outliers, situated close to each other, while all the other 12 samples in the analysis clustered together. Possibly, these two children had a deviating community composition of the gut microbiota before treatment, which was normalized during treatment with ETN, but no other findings in the present study indicated that treatment with ETN affected the gut microbiota. An observation is that both of these children were boys diagnosed with enthesitis-related arthritis, a category of JIA that is considered to exhibit a different microbial community composition than that in healthy children [3, 6, 33], although recently published data from our group does not support such a difference [34]. It would have been interesting to compare the effect of treatment on different JIA categories, but our study cohort was too small for this. Children with JIA treated with ETN have been found to have an increased risk for IBD [16], and since changes in the gut microbiota have been associated with the development of IBD [35], it is important to further examine the possible effect of ETN treatment on gut microbiota in a larger study.

In addition to analyzing the composition of microbiota, it is important to consider the function of the microbiota and its potential impact on the inflammation in JIA. We chose to analyze fecal SCFAs in pairwise comparisons, but we did not find any significant differences in the amount of the dominant SCFAs during treatment with MTX or ETN. The only significant difference in SCFAs, before and during treatment, was a higher amount of iso-butyrate in samples collected during MTX treatment, compared with samples collected before any medication. An increase in iso-butyrate has been seen in people with constipation [36], but does not, to our knowledge, have any known association to rheumatic diseases or other autoimmune diseases. It constitutes only a very small part of the total amount of SCFAs and the concentrations were very low in all samples, both before and during treatment. Also, the standard errors were large in relation to the mean values. In summary, this makes it highly doubtful that this finding is associated with MTX treatment.

Some studies on humans have indicated that NSAIDs could affect the gut microbiota [37, 38], and no correction for the use of NSAIDs was made in the present study. However, previous studies included few participants and did not correct for confounding factors such as age and medical conditions [37, 38]. In the present study, the fecal microbiota in children with JIA treated continuously with NSAIDs as the only anti-inflammatory drug was compared with that in untreated children with JIA and no significant differences were found.

One limitation in this study was the low number of children during treatment, especially children treated with ETN as a monotherapy. Since only four children had single treatment with ETN, it was not possible to draw any comprehensive conclusions regarding if ETN affects the fecal microbiota. In the study by Picchianti-Diamanti et al., several significant changes were found in patients receiving ETN as single treatment, but no changes were observed in patients receiving a combination of ETN and MTX [18]. To further investigate the effect of ETN on fecal microbiota, a study with a larger number of children treated with ETN as single treatment is needed. Also, we did not include any healthy control group in the present study, which is a limitation since it would have been interesting to know if the gut microbiota in the examined cohort of JIA patients was different than that in healthy children before treatment. However, a recent study including most of the treatment-naïve children in the present study did not show any significant differences in gut microbiota composition between untreated children with JIA and healthy children [34].

Another limitation is that we did not use any questionnaire regarding diet or probiotics for the children included in the study. Diet has a well-known impact on the gut microbiota and differences in diet could have affected the results and may have been a factor influencing the change in microbiota in some of the paired samples [39]. This confounding factor is probably a minor limitation for the paired comparisons.

The fecal samples were collected at home and stored at + 4 °C for up to 60 h before they were put in a freezer. This is a limitation, as the microbiota composition may change during that time. However, a previous study on the stability of fecal microbiota showed that the composition of microbiota did not change significantly during postal delivery of fecal samples to a laboratory [40]. The composition of microbiota seemed to be relatively stable for at least 2 days in room temperature [40].

## Conclusion

This study on children with JIA shows minor changes in the composition of microbiota during treatment with MTX or ETN, but no significant or consistent changes, either on the composition of microbiota or on the levels of SCFAs, suggesting that these changes were not related to the therapeutic effects of MTX or ETN. With increasing knowledge about the role of the gastrointestinal canal and gut microbiota in health and disease, it is important to investigate a possible impact on microbiota from these potent medications.

## Data Availability

The datasets used and/or analyzed during the current study are available from the corresponding author on reasonable request.

## Abbreviations

ANOSIM: Analysis of similarity
bDMARDs: Biological disease-modifying anti-rheumatic drugs
DMARDs: Disease-modifying anti-rheumatic drugs
ETN: Etanercept
IBD: Inflammatory bowel disease
JIA: Juvenile idiopathic arthritis
MTX: Methotrexate
NSAID: Non-steroid anti-inflammatory drug
PCoA: Principal coordinate analysis
PPI: Proton-pump inhibitor
RA: Rheumatoid arthritis
SCFAs: Short-chain fatty acids
TNF-α: Tumor necrosis factor alpha
